# Evaluation of Tp-e interval, Tp-e/QT ratio and index of cardiac-electrophysiological balance in patients with tinnitus

**DOI:** 10.1186/s12872-021-02227-8

**Published:** 2021-08-30

**Authors:** Hakan Kaya, Arif Suner, Mehtap Koparal, S. Cem Yucetas, Safiye Kafadar

**Affiliations:** 1 Department of Cardiology, Adiyaman Training and Research Hospital, Adiyaman, Turkey; 2 Department of Otorhinolaryngology, Adiyaman Training and Research Hospital, Adiyaman, Turkey; 3 Department of Neurosurgery, Adiyaman Training and Research Hospital, Adiyaman, Turkey; 4 Department of Radiology, Adiyaman Training and Research Hospital, Adiyaman, Turkey

**Keywords:** Autonomic nervous system, Tinnitus, Ventricular arrhythmia

## Abstract

**Background:**

Tinnitus is a common auditory symptom. Dysfunction in the autonomic nervous system (ANS) is an essential part of the etiopathogenesis of tinnitus. ANS regulates heart rate and heart rhythm and can lead to ventricular repolarization changes, which can cause malignant ventricular arrhythmias. T wave peak-to-end T(p-e) interval and T(p-e)/QT ratio are known ventricular arrhythmia indexes, and the index of cardiac-electrophysiological balance (iCEB) is a novel index that can be used to predict the risk of malignant ventricular arrhythmia. The goal of the study was to investigate these ventricular arrhythmia indexes in patients with tinnitus.

**Methods:**

The study population consisted of 240 patients with tinnitus and 240 healthy subjects. A standard 12-channel surface electrocardiogram was applied to both groups. T(p-e) interval, QT interval and QRS duration were determined. Corrected QT (QTc) was determined via Bazett’s formula. To predict ventricular arrhythmia, iCEB (QT/QRS), T(p-e)/QT, corrected iCEB (QTc/QRS) and T(p-e)/QTc values were determined and compared between groups.

**Results:**

Compared to the control group, QT (376.46 ± 36.54 vs 346.52 ± 24.51 ms), QTc (426.68 ± 24.68 vs 390.42 ± 24.04 ms), T(p-e) (75.86 ± 14.68 vs 62.42 ± 8.64 ms), T(p-e)/QT (0.201 ± 0.06 vs 0.180 ± 0.01) and T(p-e)/QTc (0.177 ± 0.06 vs 0.159 ± 0.02) were significantly higher in patients with tinnitus (p < 0.001 for all). QT/QRS (3.92 ± 0.68 vs 3.56 ± 0.32) and QTc/QRS (4.44 ± 1.03 vs 4.01 ± 0.64) were also significantly higher in patients with tinnitus (p = 0.018 and p = 0.008, respectively). In addition, significant positive correlations were found between T(p-e), T(p-e)/QTc ratio and disease duration (r = 0.792, p < 0.001; r = 0.500, p < 0.001, respectively).

**Conclusion:**

As a result, patients with tinnitus may have an increased risk of malignant ventricular arrhythmia.

## Background

Tinnitus is one of the most common otological symptoms [1]. It is known as the sensation of sound in the ear without an outer audible stimulus and is usually classified according to the duration of the disease. For example, tinnitus is classified as acute when the condition duration is less than three months, and chronic when it is more than three months [2]. The mechanisms of cochlear and stress have been found to be involved in the etiopathogenesis of tinnitus [3]. The association between stress and tinnitus has been reported in a previous study, and stress is considered a risk factor for tinnitus [4]. The sympathetic branch of the autonomic nervous system (ANS) is related to stress, and its chronic form can also affect tinnitus [5]. A previous study suggested a positive correlation between sympathetic tone and distress related to tinnitus. In the same study, a negative correlation between parasympathetic tone and distress related to tinnitus was found [6]. Chronic tinnitus may be associated with chronic stress due to dysfunction of autonomic regulation [7].

ANS regulates the heart rate and heart rhythm. Dysfunction in the ANS might provoke alterations in ventricular repolarization and increase the risk of malignant ventricular arrhythmias. [8]. Many electrocardiogram (ECG) parameters have been used to predict ventricular arrhythmias. For instance, T wave peak-to-end T(p-e) is used as an index of the transmural dispersion of repolarization (TDR), while T(p-e)/QT ratio includes values of the transmural and spatial dispersion of repolarization. TDR is a potential indicator for ventricular arrhythmias [9–11]. Recently, the index of cardiac-electrophysiological balance (iCEB), which is calculated as QT interval divided by QRS duration (QT/QRS), was identified as a potential marker for anticipating drug-induced ventricular arrhythmias in an animal model [12]. Multiple studies suggested that iCEB can be used as a surrogate of cardiac wavelength, which in turn is an essential factor for ventricular arrhythmias susceptibility but can only be measured via invasive electrophysiology. It has been reported that iCEB might be a noninvasive and quickly assessable marker to identify the raised risk of arrhythmic in patients [13]. The goal of the present study was to evaluate ventricular arrhythmia indexes in patients with tinnitus.

## Methods

### Study population and design

This study was a single-centre prospective study. The study was conducted in the Department of Otorhinolaryngology-Head and Neck Surgery and the Department of Cardiology. The study was approved by the Adıyaman University Clinical Research Ethics Committee (approval number: 2021/01-27). Written informed consent was obtained from all the participants included in the study, and they were informed that participation was voluntary and they were free to withdraw from the research. The study was carried out according to the Helsinki Declaration. Adult outpatients referred to the Department of Otorhinolaryngology-Head and Neck Surgery for chronic subjective tinnitus in one ear or both ears participated in this study. The disease duration was recorded for each patient. The control group included healthy subjects who were admitted to the Cardiology Department for cardiological checkups. A total of 240 adult patients (above 18 years) with chronic tinnitus were included in the study, while the control group had 240 healthy volunteers who were above 18 years. Demographic information and blood pressure-related values were obtained from all the participants. Other measured variables included BMI (weight in kilograms divided by height in square meters), smoking status and creatinine, blood glucose, electrolyte and thyroid-stimulating hormone levels. Blood samples were collected during the participants’ first clinical visit. Patients with left or right branch bundle block confirmed by electrocardiography, thyroid dysfunction, rheumatoid heart disease, cardiac valvular disease, chronic pulmonary disease, anaemia, electrolyte disorder, liver disease, acute or chronic infection, renal disease, atrioventricular conduction disorder, nonsinus rhythm confirmed by electrocardiography, systemic autoimmune disease as well as those using antidepressant, antipsychotic, antihistaminic or antiarrhythmic drugs were excluded from the study. In addition, patients with acute or stable coronary arterial disease, diabetes and hypertension and those using any drug that could affect sympathovagal activity were excluded from the study.

### Electrocardiographic and echocardiographic evaluation

All the participants underwent a 12-lead ECG (Nihon Kohden, Tokyo, Japan) while resting in the supine state. The ECG was set to the paper speed of 50 mm/s and calibrated such that 10 mm equals 1 mV. During the ECG recordings, all the participants had sinus rhythm. ECG data were used to assess the resting heart rate. ECG measurements of QRS duration, Tp-e intervals and QT intervals were calculated. Measurement errors were decreased by using a magnifying glass. Lead II and lead V5 was used for the measurements. The QT interval was named as the beginning of the QRS complex to the end of the T wave. The QT interval was corrected for heart rate via the Bazett formula: QTc = QT√(R-R interval) [14]. Using these measurements, the values of the T(p-e)/QT, T(p-e)/QTc, QT/QRS (iCEB) and QTc/QRS (corrected iCEB [iCEBc]) were assessed.

A Vivid 5 (General Electric, Horten, Norway) was used for transthoracic echocardiography examination with a 2.5 MHz transducer. The patients were made to lie on their left side to perform the imaging process using standard techniques according to the guidelines [15] and were monitored throughout the imaging process. The Simpson method was used to calculate the left ventricle ejection fraction (LVEF) [16].

### Statistical analysis

For the statistical analyses, SPSS 22.0 software program (SPSS Inc., Chicago, IL, USA) was used. Continuous variables were presented as mean ± standard deviation and categorical variables as numbers and percentages. Independent sample t-tests were used to compare continuous variables, while the Mann–Whitney U test was used for the noncontinuous parametric variables. The distribution of the data was evaluated using the Kolmogorov–Smirnov test. Categorical variables were compared within the study group using the chi-square test. Pearson’s correlation test was used for correlational analysis, and p-value < 0.05 was considered statistically significant.

## Results

The present study consisted of 240 patients with tinnitus (144 males; mean age = 47.82 ± 8.53 years) and 240 healthy controls (156 males; mean age = 46.54 ± 5.32 years). The clinical characteristics, echocardiographic and laboratory data of the participants are presented in Table 1. There were no significant differences in terms of gender, age, smoking status, BMI, diastolic and systolic blood pressure, LVEF and creatinine, potassium, calcium, magnesium, fasting glucose and thyroid-stimulating hormone levels between the two groups (p > 0.05 for all).

**Table 1:** Clinical characteristics, laboratory and echocardiographic findings of the groups

	Patients with tinnitus (n = 240)	Control group (n = 240)	p-value
Age (years)	47.82 ± 8.53	46.54 ± 5.32	0.137
Gender, male, n %	144 (60)	156(65)	0.538
BMI (kg/m^2^)	26.73 ± 2.34	25.82 ± 2.68	0.098
Smokers, n, (%) Disease duration (years)	96 (40) 5.32 ± 2.4	84 (35)	0.528
Systolic BP (mmHg)	124.62 ± 5.23	123.46 ± 4.65	0.812
Diastolic BP (mmHg)	72.64 ± 4.04	71.43 ± 5.06	0.526
IVS (mm)	10.52 ± 0.68	10.21 ± 0.54	0.396
PW (mm)	8.22 ± 1.23	8.04 ± 1.12	0.256
LVEF (%)	61.46 ± 3.27	61.87 ± 2.86	0.196
Creatinine (mg/dL)	0.86 ± 0.23	0.83 ± 0.21	0.364
Potassium (mmol/L)	4.32 ± 0.21	4.26 ± 0.41	0.498
Calcium (mg/dL)	9.62 ± 0.46	9.70 ± 0.48	0.594
Magnesium (mg/dL)	2.01 ± 0.32	2.12 ± 0.28	0.232
Fasting glucose (mg/dL)	98.74 ± 11.64	92.72 ± 12.23	0.168
Thyroid stimulating hormone (µU/mL)	2.32 ± 1.21	2.58 ± 1.14	0.065

*BMI* body mass index, *BP* blood pressure, *IVS* inter ventricular septum, *PW* posterior wall, *LVEF* left ventricle ejection fraction

**Fig. 1:**
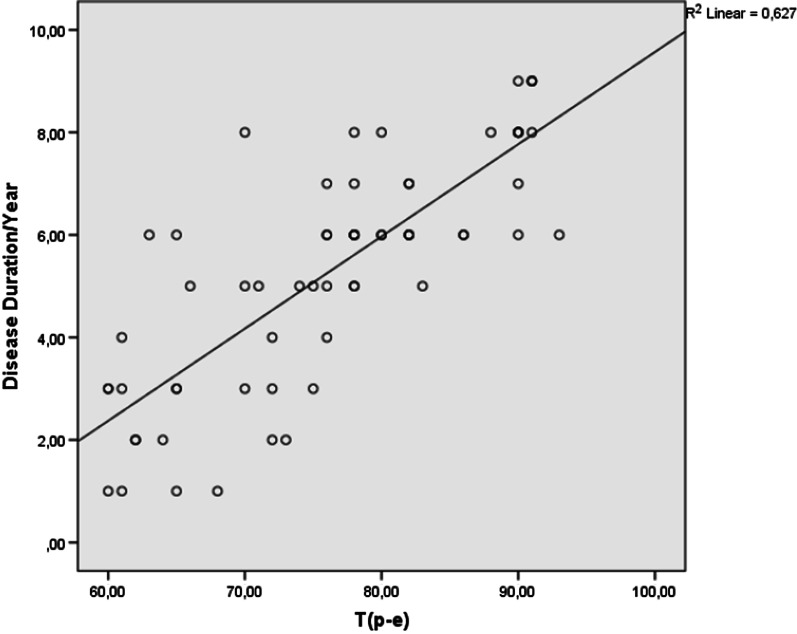
Correlations between T(p-e) interval and disease duration

**Fig. 2:**
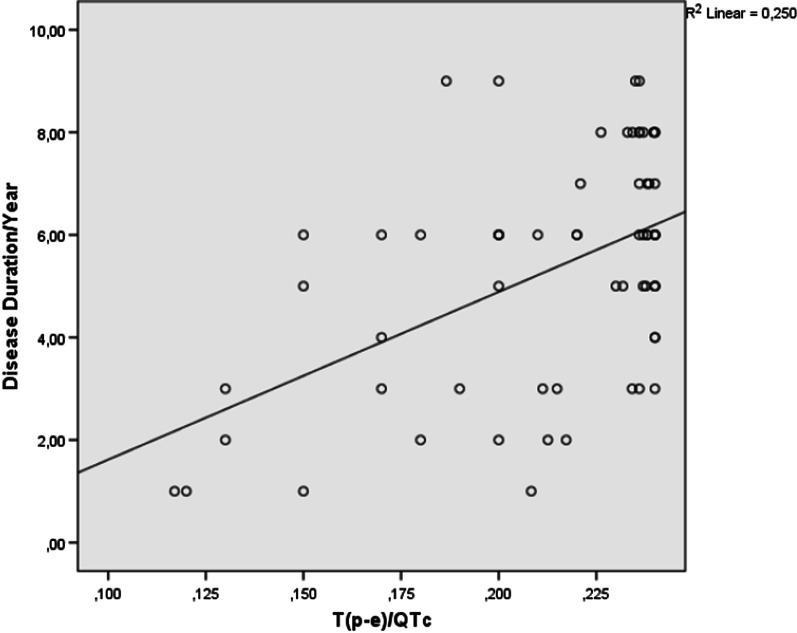
Correlations between T(p-e)/QTc ratio and disease duration

The electrocardiographic findings of the two groups are presented in Table 2. The mean heart rate and QRS duration were similar between the patients and the control group (76.24 ± 10.02 vs 77.32 ± 10.26 beats/min, p = 0.624; 96.04 ± 18.72 vs 97.24 ± 12.32 ms, p = 0.565, respectively). Compared to the control group, QT (376.46 ± 36.54 vs 346.52 ± 24.51 ms), QTc (426.68 ± 24.68 vs 390.42 ± 24.04 ms), T(p-e) (75.86 ± 14.68 vs 62.42 ± 8.64 ms), T(p-e)/QT (0.201 ± 0.06 vs 0.180 ± 0.01) and T(p-e)/QTc (0.177 ± 0.06 vs 0.159 ± 0.02) were higher in patients with tinnitus (p < 0.001 for all). QT/QRS (3.92 ± 0.68 vs 3.56 ± 0.32) and QTc/QRS (4.44 ± 1.03 vs 4.01 ± 0.64) were significantly higher in patients with tinnitus (p = 0.018 and p = 0.008, respectively). A significant positive correlation was found between T(p-e) and disease duration (r = 0.792, p < 0.001) (Fig. 1). In addition, a significant positive correlation was found between T(p-e)/QTc ratio and disease duration (r = 0.500, p < 0.001) (Fig. 2).

**Table 2:** Electrocardiographic findings of the groups

	Patients with tinnitus (n = 240)	Control group (n = 240)	p-value
Heart rate (beats/min)	76.24 ± 10.02	77.32 ± 10.26	0.624
QRS (ms)	96.04 ± 18.72	97.24 ± 12.32	0.565
QT interval (ms)	376.46 ± 36.54	346.52 ± 24.51	< 0.001
QTc interval (ms)	426.68 ± 24.68	390.42 ± 24.04	< 0.001
Tp-e interval (ms)	75.86 ± 14.68	62.42 ± 8.64	< 0.001
Tp-e/QT ratio	0.201 ± 0.06	0.180 ± 0.01	< 0.001
Tp-e/QTc ratio	0.177 ± 0.06	0.159 ± 0.02	< 0.001
iCEB (QT/QRS)	3.92 ± 0.68	3.56 ± 0.32	0.018
iCEBc (QTc/QRS)	4.44 ± 1.03	4.01 ± 0.64	0.008

*QTc* corrected QT interval, *Tp-e* T wave peak-to-end, *iCEB* index of cardio-electrophysiological balance, *iCEBc* corrected iCEB

## Discussion

In the present study, QT, QTc, T(p-e), T(p-e)/QT, T(p-e)/QTc, iCEB and iCEBc parameters were significantly higher in patients with tinnitus when compared to the control group. In addition, significant positive correlations were found between T(p-e), T(p-e)/QTc ratio and the disease duration. These results point out a potential increased risk of malignant ventricular arrhythmias in patients with tinnitus. To the best of our knowledge, no study has examined these parameters to evaluate the risk of malignant ventricular arrhythmias in patients with tinnitus in the literature.

Tinnitus is known as the sensation of sound in the ear without an outer audible stimulus [17]. The etiopathogenesis of tinnitus is not only associated with the cochlear mechanisms but also with stress. Current pathophysiological models link the initiation and maintenance of tinnitus with stress [18, 19]. In addition, studies have shown that stress is an effective factor that can turn mild tinnitus into a severe one [20]. The sympathetic branch of the ANS plays a major role in stress [21]. ANS also plays a major role in the development of ventricular arrhythmias, as it is a major modulator of ventricular repolarization. Several mechanisms linking ANS dysfunction and ventricular arrhythmias are very well established, albeit some details are still to be unravelled [22]. Methods such as hormonal mediator tests, pharmacological tests and various psychological stress tests could be used to evaluate ANS functions. One of these methods is heart rate variability (HRV), which is controlled by sympathetic and parasympathetic cardiac nerves. While sympathetic activity increases heart rate, parasympathetic activity decreases heart rate. HRV studies have also improved our knowledge of the effects of physiological events on tinnitus. It has been found that parasympathetic activity and sympathetic activity can be assessed using HRV parameters. The association between ANS dysfunction and cardiac autonomous dysfunction has been investigated in many studies through using HRV. A previous study showed an increase in sympathetic activity in patients with rheumatoid arthritis using HRV parameters and reported that this might cause malignant ventricular arrhythmias [23]. Patients with systemic lupus erythematosus have been found to have abnormal HRV, which reflected increased sympathetic activity and cardiac autonomic dysfunction [24]. Choi et al. evaluated HRV parameters in patients with tinnitus and demonstrated that sympathetic activity increased in these patients [25]. They also observed a positive correlation of the tinnitus period with sympathetic activity [25]. Another study that investigated the relationship between neural activity and the severity of tinnitus showed a positive correlation between sympathetic markers and tinnitus distress, and a negative correlation between tinnitus distress and parasympathetic markers [26]. Betz et al. observed that HRV parameters were similar between patients with tinnitus and the healthy control group. However, subjectively, they reported an increased level of tinnitus after exposure to stress in the same study [27]. HRV measurements are complex and quite different, as different researchers measure different time units, evaluation and comparison using the obtained data may give inaccurate outcomes [28].

T wave represents ventricular repolarization at ECG. T(p-e) interval has been used as an index of the TDR. HRV assesses the sympathetic-parasympathetic balance at the sinus node level, while measures of ventricular repolarization are influenced, among other factors, by the sympathetic-parasympathetic balance at the ventricular level. Parameters such as T(p-e)/QT and T(p-e)/QTc ratios, which are sensitive, have recently been used to predict ventricular repolarization dispersion [29, 30]. An increase in T(p-e)/QT ratio and T(p-e) interval has been reported in various cardiac diseases such as Brugada, long QT and short QT syndromes, and noncardiac diseases such as autoimmune hepatitis, psoriasis and obstructive sleep apnoea [30–33]. Lecca et al. reported that QTc was prolonged among workers operating at a site where stress level was found to be elevated and suggested that work-related stress had subclinical effects on the cardiac function autonomic regulation [34]. Afsin et al. indicated that the T(p-e)/QT ratio and T(p-e) interval increased among patients with panic disorder and suggested that this might be due to the dysfunction of ANS through elevated sympathetic activity [35]. Similarly, it has been reported that T(p-e) and T(p-e)/QT values increased in patients with gastroesophageal reflux disease, which might be due to increased sympathetic activity [36]. A previous study reported that tinnitus is associated with sleep deprivation, emotional difficulties and social interaction problems [37]. A positive correlation has been reported between sleep deprivation and T(p-e)/QT and T(p-e) values [38]. Our results showed a significant increase in T(p-e) and T(p-e)/QT values in patients with tinnitus and this might be due to the dysfunction of ANS through elevated sympathetic activity, which is consistent with previous studies.

iCEB is a noninvasive parameter used to estimate the risk of ventricular proarrhythmia. iCEB may offer knowledge for both repolarization and depolarisation of the cardiac action potential. Such a feature may estimate the cardiac proarrhythmia risk better than T wave with repolarization only, TDR and QT interval variability. There are few studies on iCEB in the literature. Nafakhi et al. demonstrated that an increase in iCEB was related to the raised pericardial fat volume in patients with suspected coronary artery disease [39]. Yumurtaci et al. demonstrated that patients with acute myocarditis who had arrhythmia had higher levels of iCEB, T(p-e), T(p-e)/QTc and T(p-e)/QT than those without arrhythmia [40]. Our study showed that iCEB and iCEBc values were significantly higher in patients with tinnitus compared to the control group. In our study, the larger values of iCEB and iCEBc observed in the patients versus the controls mostly reflect the longer QTc value (no differences were found in QRS duration).

### Study limitations

The major limitations of the present study are the small number of samples, the use of only one study centre and the lack of follow-up of patients for ECG changes, sudden cardiac death or malignant ventricular arrhythmia.

## Conclusion

Our result revealed that QT, QTc, T(p-e), T(p-e)/QT, T(p-e)/QTc iCEB and iCEBc values were significantly higher in patients with tinnitus compared to the healthy group. In addition, significant positive correlations were found between T(p-e), T(p-e)/QTc ratio and disease duration. According to our results, patients with tinnitus may have an increased risk of malignant ventricular arrhythmias. Further studies with follow-up are needed to validate our findings.

## Data Availability

The datasets used and analysed during the current study are available from the corresponding author upon reasonable request.

## Abbreviations

ANS: Autonomic nervous system
BMI: Body mass index
T(p-e): T wave peak-to-end
iCEB: Index of cardiac-electrophysiological balance
iCEBc: Corrected index of cardiac-electrophysiological balance
QTc: Corrected QT interval
ECG: Electrocardiogram
TDR: Transmural dispersion of repolarization
LVEF: Left ventricle ejection fraction
HRV: Heart rate variability
BP: Blood pressure
IVS: Interventricular septum
PW: Posterior wall
